# Three Variants Affecting Exon 1 of *Ectodysplasin A* Cause X-Linked Hypohidrotic Ectodermal Dysplasia: Clinical and Molecular Characteristics

**DOI:** 10.3389/fgene.2022.916340

**Published:** 2022-07-06

**Authors:** Yupei Wang, Chuan Zhang, Bingbo Zhou, Ling Hui, Lei Zheng, Xue Chen, Shifan Wang, Lan Yang, Shengju Hao, Qinghua Zhang

**Affiliations:** ^1^ Medical Genetics Center, Gansu Provincial Maternity and Child-care Hospital, Lanzhou, China; ^2^ Gansu Provincial Clinical Research Center for Birth Defects and Rare Diseases, Lanzhou, China

**Keywords:** hypohidrotic ectodermal dysplasia, EDA, whole-exome sequencing, splicing variant, cryptic splice site, HED, ectodysplasin A

## Abstract

**Background:** Ectodysplasin A (EDA) variations are major pathogenic factors for hypohidrotic ectodermal dysplasia (HED), the most common form of ectodermal dysplasia (ED), characterized by hypotrichosis, hypohidrosis, hypodontia, and other oral features.

**Methods:** Molecular genetic defects in three HED families were detected by whole-exome sequencing and confirmed by Sanger sequencing or multiplex ligation-dependent probe amplification. The effect of splicing variant was further verified by EDA minigene *in vitro* analysis. De novo deletion was confirmed by chromosomal microarray analysis.

**Results:** Three variants (c.396 + 1 G > C, c.171-173 del GTT, and exon 1 deletion) were identified, all affecting exon 1 of the *EDA* gene. Variants c.396 + 1 G > C and c.171-173 del GTT were first identified. Minigene analysis of the splicing variant (c.396 + 1 G > C) displayed a prolonged EDA-A1 transcript containing extra 699 bp at the start of intron 1, representing a functional cryptic splice site formation *in vitro*. Combining the results of chromosomal microarray analysis and whole-exome sequencing, the deletion variant was over 87 kb. Three variants were predicted to affect protein function to differing degrees, and were responsible for X-linked HED with varying phenotype.

**Conclusion:** Investigating the clinical and molecular characteristics of these variations broadens our understanding of *EDA* gene variants, supporting clinical diagnosis, genetic counseling, and prenatal diagnosis of HED.

## Introduction

Ectodermal dysplasia (ED) is a set of genetic diseases with two or more abnormally developed ectoderm-derived structures, including hair, teeth, nails, skin, and sweat glands (Pigno et al., 1996; Vaidya et al., 2013; Wright et al., 2019). Of the 200 different types of ED reported, ∼30 causative genes have been identified at the molecular level (Itin and Fistarol, 2004). Genetic variations in ectodysplasin A (EDA) pathway genes, such as *EDA*, ectodysplasin A receptor (*EDAR*), and EDAR-associated adapter protein (*EDARADD*) are known to be associated with hypohidrotic ED (HED); the prevalence of which is ∼1/100,000 (Khabour et al., 2010; Deshmukh and Prashanth, 2012; Okita et al., 2019). *EDA* is a unique gene involved in the pathogenesis of X-linked hypohidrotic ectodermal dysplasia (XLHED; OMIM 305100), which accounts for 95% of patients with HED, and the remaining 5% are mainly due to autosomal dominant or recessive inheritance (Clarke et al., 1987; Clauss et al., 2008; Deshmukh and Prashanth, 2012; Wang et al., 2020). XLHED is characterized by a clinical triad of hypodontia (congenital absence of teeth), hypoplasia of sweat glands, and hypotrichosis (sparse hair) (Moura et al., 2019).

The *EDA* gene is located on chromosome Xq12-q13.1, which has nine exons, and consists of four domains: a transmembrane domain, furin recognition sequences, a collagen domain, and β-sheets A−H of the tumor necrosis factor (TNF) homology domain, encoded by exon 1, 3, 5–6 and 7–9, respectively (Schneider et al., 2001). It was identified as a membrane-bound signaling molecule of the TNF superfamily (Trzeciak and Koczorowski, 2016). Variations in the *EDA* gene lead to loss or dysfunction of EDA1, associated with the signaling of the epithelial–mesenchymal transition during embryogenesis, as well as the initiation and development of ectodermal derivatives (Korber et al., 2020). At least 100 variants of the *EDA* gene have been identified as pathogenic mutations in the NCBI ClinVar database (http://www.ncbi.nlm.nih.gov) based on published papers and submissions (Liu et al., 2012; Huang et al., 2015).

In the present study, we identified three variants of the *EDA* gene in three Chinese families with XLHED, and demonstrated that variants led to varying clinical phenotypes through different molecular mechanisms affected by exon 1 of the *EDA* gene.

## Materials and Methods

### Case Information

Patients and their parents attended the Medical Genetics Centre, Gansu Provincial Maternity, and Childcare Hospital (Lanzhou, China), seeking genetic diagnosis of congenital symptoms (no sweating, pyrexia, and dysplasia of hair and teeth). Proband 1 was a 30-year-oldman; proband 2 was a 10-month-old boy; and proband 3 was a 20-day-old boy. Their parents were healthy and unrelated. All participants gave their signed informed consent for genetic studies before collecting blood samples or performing clinical evaluations.

### DNA Extraction

Peripheral blood (3–5 ml) was collected from proband family members for DNA extraction using a Genomic DNA Extraction kit (Tiangen Biotech, Beijing, China), and extracted genomic DNA was subsequently used for targeted whole-exome sequencing (WES) and Sanger sequencing.

### Whole-Exome Sequencing

Trio WES was carried out by MyGenostics Co., Ltd (Beijing, China). Briefly, qualified genomic DNA was fragmented randomly to an average size of 180 bp with a Bioruptor sonicator (Diagenode, Liege, NJ, United States). The fragmented DNA was then repaired and A-tails were ligated to the 3' end. Next, Illumina adapters (Illumina Inc., United States) were ligated to the fragments, and adapted DNA templates were amplified by PCR. DNA was then captured using a GenCap Custom Enrichment kit (MyGenosticsGenCap Enrichment Technologies, Beijing, China) and sequenced on an Illumina HiSeq 2500 platform (Illumina Inc.) as paired-end 90 bp reads. The mean sequencing depth was >100. N20 reads covered targeted bases by >95%.

### Bioinformatics Analysis

Using the Trim Galore program, reads of low quality and adapters were filtered out after sequencing. SOAP aligner (SOAP v2.21) was used to align clean reads to the h19 human reference genome (UCSC Genome Browser hg19). Insertions, deletions, and single-nucleotide polymorphisms (SNPs) were identified by the Burrows–Wheeler alignment program (0.7.12-r1044) and tested using a GATK tool kit. The exome assistant program was used to annotate the locations of exonic, intronic, and intergenic regions, as well as protein-coding effects such as synonymous, missense, nonsense, and frameshift (Xu et al., 2015). Frequency and function were the main factors used to obtain candidate variants for further analysis. For the frequency filter, a 0.01 cut-off was applied according to allele frequency estimates from NCBI dbSNP (v152; http://www.ncbi.nlm.nih.gov/projects/SNP/), 1,000Gome (http://www.ncbi.nlm.nih.gov/Ftp/), and Exome Aggregation Consortium (http://gnomad.broadinstitute.org/) databases. Synonymous and missense variants, which were predicted to be benign or tolerated in Sorting Intolerant From Tolerant (SIFT), PolyPhen-2, Mutation Taster, and GERP++, were removed by the functional filter. Splicing variants were evaluated by MaxEntScan and dbscSNV11.

### Sanger Sequencing Validation

In order to identify the target variants associated with HED in family members, Sanger sequencing was performed. Direct PCR products were sequenced using BigDye Terminator v3.1 Cycle Sequencing kits (Applied Biosystems, Foster City, CA, United States) and analyzed on an ABI3500DX Genetic Analyzer (Applied Biosystems, Warrington, United Kingdom) using *EDA* primers (pedigree 1, Forward, 5′-act​cca​ctc​tga​ctc​cca​gga​c-3’; Reverse, 5′-ctg​gtc​ctg​ccc​tct​aaa​ttg-3’; pedigree 2, Forward, 5′-gcc​tca​aga​gag​tgg​gtg​tc-3’; Reverse, 5′-gtc​ctg​gga​gtc​aga​gtg​ga-3′).

### Minigene Construct Generation, Transfection, and RT-PCR

In order to further investigate the pathogenic mechanism of the splicing variant at the 5’ (donor) splice site (5’ss; c.396 + 1 G > C), a minigene containing exon 1, partial intron 1, and exon 2 of the *EDA* gene were designed using exon-trapping pEGFP-C1 plasmids. Specifically, exon 1, the first and last 1,000 bp of intron 1 (the intermediate sequence of intron 1 was deleted), and exon 2 encoding wild-type (WT) or mutant type (MT) sequences were incorporated into the pEGFP-C1 vector within the 5′ *Xho*l and 3′ *Bam*HI restriction enzyme sites using specific primers. WT and MT expression vector construction was performed by Hitrobio Tech, Beijing, China. All recombinant plasmids were validated by direct sequencing. Human embryonic kidney 293T (HEK 293T) cells were grown in Dulbecco’s modified Eagle medium (DMEM) supplemented with 10% fetal bovine serum (Thermo Fisher Scientific, MA, United States), penicillin (100 U/L), and streptomycin (100 mg/L) at 37°C with 5% CO_2_. Transfection was performed using HEK 293T cells grown to 70–80% confluence in the serum-free medium by Lipofectamine 2000 Reagent (Thermo Fisher Scientific) following the manufacturer’s instructions, and cells were collected at 48 h after transfection. For RT-PCR analysis, a MiniBEST Universal RNA Extraction kit (Takara, Dalian, China) was used to extract total RNA and a PrimerScript RT Reagent kit (Takara) was used for reverse transcription. PCR amplification of minigene transcripts was conducted using vector-specific forward primer pEGFP-C-5 F (5′-CAT​GGT​CCT​GCT​GGA​GTT​CGT​G-3′) and reverse primer pEGFP-C-3 R (5′-ATC​TCA​GTG​GTA​TTT​GTG​AGC-3′). PCR products were identified by 1% agarose gel electrophoresis, and Sanger sequencing was performed to analyze mutant patterns.

### Multiplex Ligation-Dependent Probe Amplification

MLPA was performed on the pedigree 3 using a SALSA MLPA Probemix P183 kit (MRC-Holland, Amsterdam, the Netherlands; http://www.mlpa.com). This kit included probes from all exons of *EDARR*, *EDAR*, *EDA*, and *WINT10A*, all associated with HED. The assay was performed according to the manufacturer’s protocol. Briefly, 100 ng genomic DNA was denatured at 98°C for 5 min then allowed to hybridize to the MLPA probe overnight. Ligation reactions were then performed using Ligase-65 enzyme and PCR was carried out with SALSA PCR primers. PCR products were separated by capillary electrophoresis on an ABI 3500 Genetic Analyzer (Applied Biosystems). Original data were analyzed by Gene mapper 4.0 and Coffalyser.Net software, and copy number was calculated according to the MLPA kit instructions.

### Chromosomal Microarray Analysis

CMA was performed with a CytoScan 750K array (Affymetrix, Santa Clara, CA, United States) according to the manufacturer’s recommendations. Genomic DNA of 40 ng/μL was digested, ligated with adaptors, amplified, purified, labeled, and then hybridized into the array. After the completion of hybridization, the array was washed with buffer, stained, and scanned with a laser scanner. Data were analyzed with Chromosome Analysis Suite (ChAS) (version 4.2.0.80) software. The hg19/GRCh37 genome was used for genomic assembly. All identified variants were further analyzed with reference to public databases including Database of Genomic Variants (DGV, http://projects.tcag.ca/variation), the 1,000 Genome Project (http://browser.1000genomes.org), DECIPHER (http://decipher.sanger.ac.uk/), gnomAD (http://gnomad.broadinstitute.org/), ClinVar (https://www.ncbi.nlm.nih.gov/clinvar/), and OMIM (https://www.omim.org).

## Results

### Clinical Examination

**FIGURE 1:**
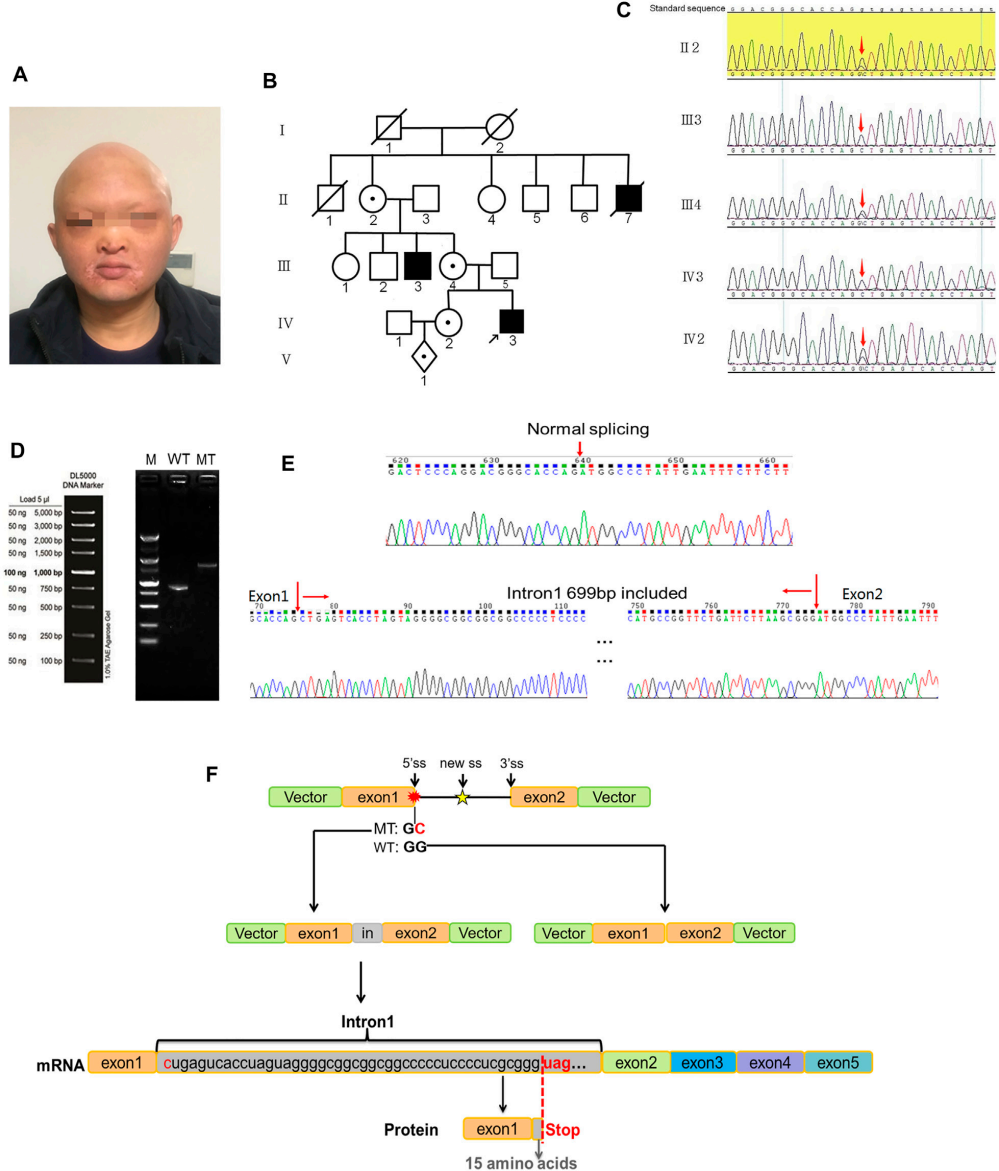
Variants in case 1. **(A)** Frontal photograph of proband 1 showing various developmental defects of ectodermal appendages including protuberant lips, short chin, and flat nose. **(B)** Family tree showing that the proband’s uncle (III3) and uncle’s grandpa (II7) were HED positive. **(C)** Validation of the mutation site by Sanger sequencing. The red arrow indicates the mutated base. **(D)** Electrophoretogram results of transcripts generated from the transfected WT and MT EDA minigenes. Lane M, 5,000 bp markers; Lane WT, WT EDA minigene transcripts showing a single band of 821 bp; Lane MT, MT minigenes containing mutant alleles showing a longer band (the empty lane has been cropped). **(E)** Inclusion of an extra 699 nucleotides in intron 1 validated by Sanger sequencing. **(F)** The splicing and transcription pattern of MT EDA pre-mRNA.

Proband 1 presented a distinctive facial appearance with sparse hair and eyelashes, and no eyebrows, along with a flat nose, thick lips, and albino around the lips (Figure 1A). His uncle (III3) and uncle’s grandpa (II7) were both HED patients presenting the same symptoms. Probands 2 and 3 showed a similar phenotype with sparse hair, dry skin, and frequent fever. As early as the fetus stage, alveolar bone dysplasia was identified in proband 3 by sonographic examination, indicating thinner upper alveolar bones and fewer tooth germs compared to normal fetus, as we reported previously (Li et al., 2021). After birth, his skin appeared abnormally dry and wrinkled (Figure 3B) and his upper alveolar bones were thinner compared with normal. Proband 2 also appeared to have thinner alveolar bone at 10 months. A preliminary diagnosis of HED was made by a dermatologist based on the clinical manifestations presented by all probands (Julia et al., 2018). The varying symptoms of the three patients are shown in Table 1.

**TABLE 1:** The genetic characteristics of *EDA* gene in patients.

No.	Age	Gene	Gender	Phenotype					Chromosome Position	Transcript/Exon	Mutation Analysis	Mutation type	MAF	SIFT	PolyPhen_2	REVEL	ACMG Classification (Evidence of pathogenicity)
				Hypodontia	Hypotrichosis	Fever	Dry Skin	Special Face									
1	30 Years	EDA	Male	+	+++	+	+	+	chrX-68836549	NM_001005609 exon1	c.396 + 1 G > C	Splicing mutation	-	unknown	benign	unknown	PVS + PM2 (likely pathogenic)
2	10 Months	EDA	Male	+	+	+	+	-	chrX-68836322–68836325	NM_001399 exon1	c.171_173 delGTT (p.57_58days elTLinsT)	Indels mutation	-	unknown	unknown	unknown	PM1+PM2+PM4+PP4 (likely pathogenic)
3	20 Days	EDA	Male	+	+++	+	+++	+	exon1	NM_001399 exon 1	del exon 1	Indels mutation	-	-	-	-	PVS1+PS1+PS2+PP4 (pathogenic)

Note: means without corresponding phenotype; + means with corresponding phenotype; +++ means with severe phenotype; MAF: minor allele frequency; ACMG: American College of Medical Genetics and Genomics; PS: pathogenic strong; PP: pathogenic supposing; PM, pathogenic moderate; PVS, pathogenic very strong.

### Identification of *EDA* Gene Variants

**FIGURE 2:**
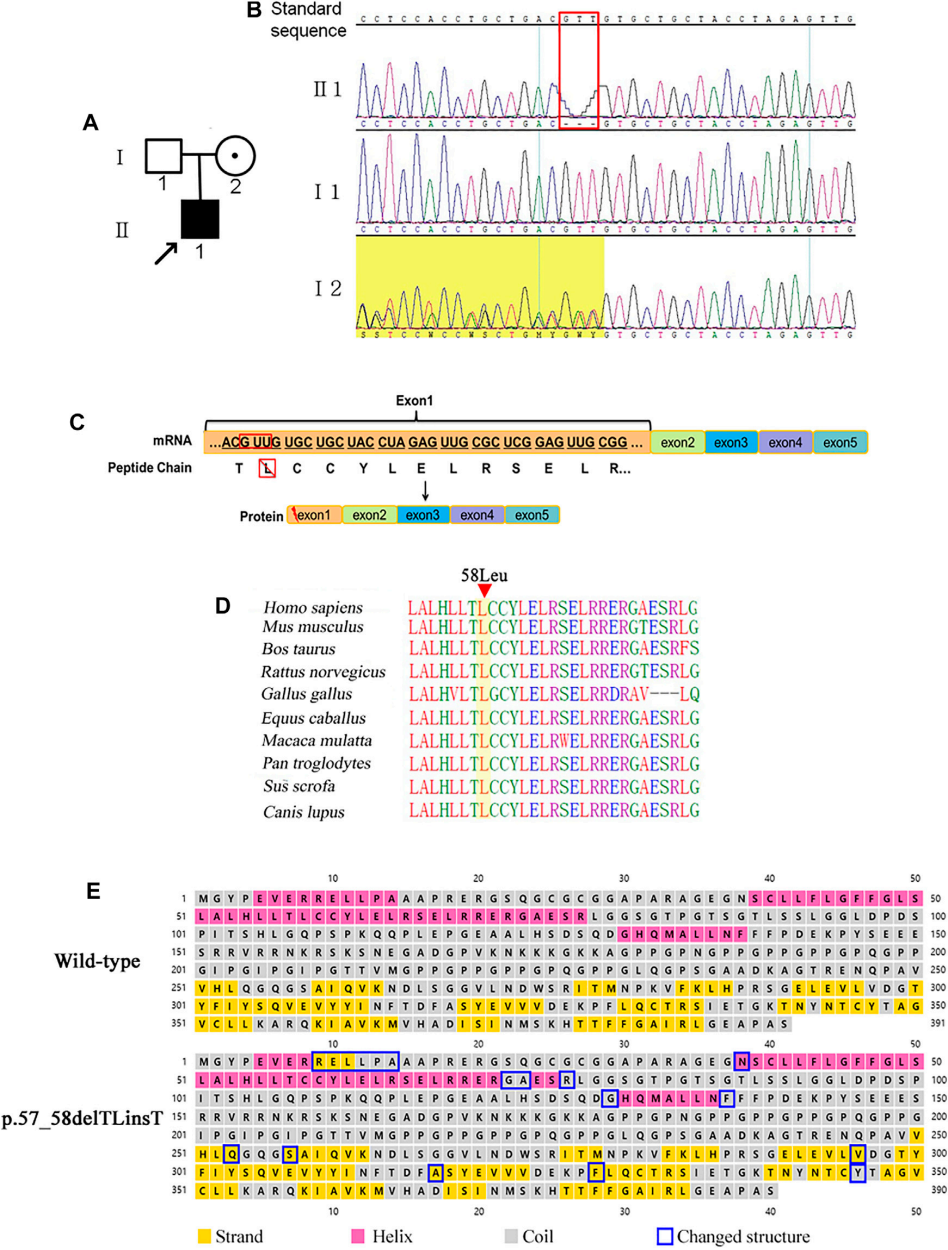
Variants in case 2. **(A)** Family tree showing the mutations inherited from I2. **(B)** Validation of the mutation site (c.171_173delGTT) by Sanger sequencing. **(C)** Transcription pattern. **(D)** Conservation analysis of affected amino acids in the EDA protein among 10 different mammalian species. **(E)** Secondary structure prediction of the WT EDA protein and the p.57_58delTLinsT mutant.

**FIGURE 3:**
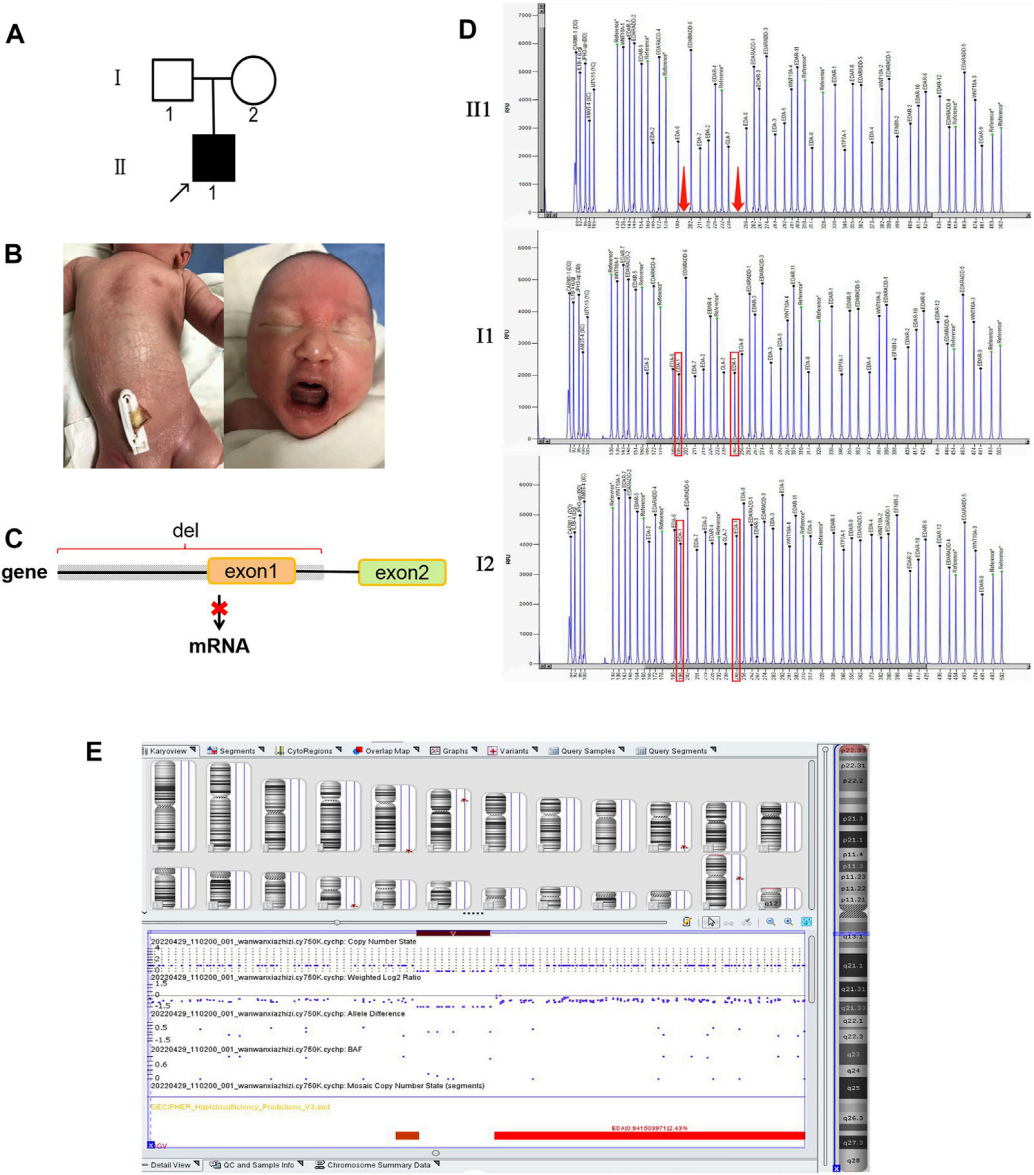
Variants in case 3. **(A)** Family tree showing a *de novo* mutation. **(B)** Dry skin of proband 3. **(C)** Transcription pattern. **(D)** MLPA results show deletion of exon 1. **(E)** CMA profile showing a loss of 82 kb of Xq (chrX: 68,831,364–68,748,640; hg19) (dark red box) which is upstream of the *EDA* gene. Images were generated using the Chromosome Analysis Suite (ChAS) software.

A hemizygous splicing variant of the *EDA* gene (c.396 + 1 G > C) was identified in pedigree 1 (Figure 1C). The variant resulted in the destruction of the splicing donor. There is no information in the 1,000 Genomes, MyGenosticsInhouse, ESP6500, EXAC, or ExAC_EAS population databases about this variant. It was predicted to be deleterious by MaxEntScan following analysis of variants near the 5′ and 3′ splice sites. Additionally, dbscSNV11 predicted that the variant was deleterious. The hemizygous variant c.396 + 1 G > C of the *EDA* gene was verified by Sanger sequencing, and it was judged to be pathogenic based on American College of Medical Genetics and Genomics (ACMG) Guidelines. Furthermore, II2, III4, and IV2 carried the same variant, while III3 was a patient and died (Figure 1B). Employing amniotic fluid puncture for prenatal diagnosis, fetus V2 was detected as a carrier with *EDA* gene heterozygous variant c.396 + 1 G > C. Similarly, proband 2 and proband 3 were identified as c.171-173 del GTT and exon 1 deletion hemizygous variants of the EDA gene respectively. Sanger sequencing was then performed on available lineage members (Figure 2B). The results showed that the variant in proband 2 (c.171-173 del GTT) was inherited from his mother (Figure 2A). However, MLPA showed the mother of proband 3 did not carry the same variant, indicating a *de novo* deletion variant (Figure 3D). Based on raw data of WES and probe coverage of MLPA, boundaries of this deletion established at the upstream and downstream regions of exon 1. The length of the deletion can be roughly estimated by CytoScan750k gene chip for its composition of 550,000 non-polymorphic CNV probes and more than 200,000 SNP probes. According to the position of the missing SNP, there is a deletion of 82,724 bp in the X chromosome (chrX: 68,748,640–68,831,364), which belong to the upstream region of EDA gene (Figure 3E). At downstream of missing SNP region, there are 6314 bp length without SNP coverage which contain upstream of exon 1, exon 1 and partial intron 1 of EDA gene. Combining the results of CMA, MLPA, and WES, the absolute deletion length extended to downstream of exon 1, which is 87,938 bp (chrX: 68,748,640–68,836,578) including upstream of exon 1 (87,513 bp) and exon 1 (396 bp), and downstream of exon 1 (29 bp). In fact, the deletion length might be longer. Detailed information for *EDA* variations is shown in Table 1. Variants c.396 + 1 G > C and c.171-173 del GTT are reported herein for the first time.

### RT-PCR Analysis of *EDA* Minigenes

RT-PCR assays were performed to investigate transcripts generated from transfected WT and MT *EDA* minigenes. The WT *EDA* minigenes produced a single band of 821 bp comprising the expected pEGFP-C-5′, exon 1, exon 2, and pEGFP-C-3′ regions. However, the MT RT-PCR products showed a longer band (Figure 1D). Sequence analysis of the abnormal products revealed the inclusion of the first 699 nucleotides from intron 1, which may be attributed to the presence of a cryptic 5′ splice site in intron 1 because the AG sequence was observed at positions 700 and 701 of intron 1 (Figure 1E).

### Protein Function Prediction

For proband 1, inclusion of the first 699 nucleotides of intron 1 could potentially result in a redundant reading frame in exon 1 of the *EDA* gene, and ultimately generated a longer peptide. However, there was a stop codon at positions c.396 + 46–48 that could lead to abnormal truncated EDA-A1 proteins or nonsense-mediated mRNA decay (NMD; Figure 2F). Therefore, c.396 + 1 G > C was judged to be a loss-of-function variant.

For proband 2, variants of c.171-173 del GTT resulted in a single amino acid deletion of the leucine codon (Figure 2C). The mutation taster tool predicted this to be a disease-causing variation. Comparative sequence alignment was performed across most mammals using the T-Coffee Multiple Sequence Alignment Program (http://www.ebi.ac.uk/Tools/msa/tcoffee). The results showed that this amino acid has been conserved in mammals during evolution, and is therefore important for protein structure and/or function (Figure 2D). Furthermore, this variation might cause changes in the α-helical and β-sheet secondary structure components of the EDA protein predicted by Psipred 4.0 software (Figure 2E).

For proband 3, MLPA was applied to confirm the absence of exon 1 of the *EDA* gene (Figure 3D). The results showed that two peaks representing exon 1 were absent in proband 3 but not in his parents. The detection of CMA indicated that there was a large fragment deletion, including the upstream region (87 kb) of the EDA gene, which completely destroyed the initiation of transcription of *EDA* and caused the failure of EDA protein synthesis (Figure 3C).

## Discussion

According to the HGMD Professional database (2017.2), there are 355 registered variations of the *EDA1* gene, of which 31 occur in the intron region. In the three HED pedigrees, an unreported 5’ss variant (c.396 + 1 G > C) of the *EDA* gene was found, and the splicing alteration mechanism was confirmed by minigene *in vitro*. The variant led to aberrant pre-mRNA splicing in exon 1 of the *EDA* gene, which generates a longer transcript with an extra 699 nucleotides in intron 1. In most cases (98.7%), GT and AG sequences are canonical splice sequences at the 5′ and 3′ ends of the intron, respectively, that define exon–intron boundaries for spliceosome recognition. The 5′ donor splice site variants at + 1 and +2 positions, as well as the 3′ acceptor splice site variants at–1 and −2 positions, are considered pathogenic (Caridi et al., 2016; Anna and Monika, 2018; Ma et al., 2019). Furthermore, variant c.526+1G > A in the *EDA* splice donor site caused the complete omission of exon 3, which resulted in abnormal truncated EDA-A1 proteins (Liu et al., 2018). In addition, c.396 + 5 G > A and c.396 + 2 T > C variants were also reported in HED cases without further research.

Splicing variants can be summarized into four types according to the impact on the final composition of mRNA: 1) Exon skipping, in which an authentic splice site variant and a variant within an exon usually result in whole-exon skipping; 2) cryptic exon inclusion, in which inclusion of a subsequent intron (pseudo exon) is caused by a deeply intronic nucleotide variant; 3) exon sequence removal, in which activation of cryptic splice sites caused by a single-nucleotide variant in an exon results in exclusion of exonic sequences; and 4) intron inclusion, in which intronic sequence inclusion generated by a variant in the intron or authentic site leads to the generation of a cryptic intronic splice site (Wimmer et al., 2007; Anna and Monika, 2018). Variants at the canonical splice sequence either result in skipping of one or more adjacent exons, or activation of a cryptic splice site of the same type in a neighboring exon site. In case 1, the positions of c.369 + 700–701 present alternative GT nucleotides as a stronger cryptic intronic splice site described in type 4, resulting in inclusion of an intron fragment instead of exon skipping. For 5’ss, a highly significant correlation was observed between the mutational consequences at the RNA level and the number of potentially utilizable cryptic donor splice sites in the region around the affected splice site (Buratti et al., 2007). More potential splice sites in the region around the affected splice site mean an increased probability of inclusion of introns (Kovacova et al., 2020). In case 1, 32 potentially utilizable GT nucleotides upstream of the chosen splice site were ignored, and only one aberrant transcript was generated in the minigene assay. Studies show that recognition of the 5’ss in a pre-mRNA is initiated by the formation of a base pairing interaction between the splice site sequence and particular sequences in the U1 snRNA of the spliceosome (Liu et al., 2017). Hence, the chosen cryptic donor splice site may be associated with a strong splicing motif for greater sequence complementarity, leading to the 5′ end of U1 snRNP binding with higher affinity.

Variant c.171-173 del GTT leads to p.57-58 del TL ins T in the *EDA* gene, predicted as unknown by PolyPhen_2, SIFT, and REVEL. In addition, the HGMD database has no relevant reports for this locus, hence it was evaluated as uncertain by ACMG. However, the symptoms of hypohidrosis, hypotrichosis, and thickness of the alveolar arch strongly support the clinical diagnosis of XLHED. This represents new pathogenic evidence for this variant. Variant with full deletion of exon 1 was first reported in Finland (Pääkkönen et al., 2001). In China, we previously reported the ultrasonic phenotype of this XLHED with thinner alveolar bone than a normal fetus. After birth, the infant’s upper alveolar bones were also thinner compared with normal. Hence, it is important to take the thickness of the alveolar arch into consideration for investigating intrauterine or infancy dental development.

Although all three variants affected exon 1, the results were different between variants, as we predicted. For proband 1, there was an extra stop codon in intron 1 that could lead to NMD and decreased expression of the abnormal truncated EDA-A1 protein. For proband 2, the variant led to loss of leucine in exon 1, which could affect the transmembrane transportation domain of the EDA-A1 protein. For proband 3, the variant led to loss of exon 1 and upstream region, which completely destroyed the initiation of transcription and translation. The wild-type EDA protein could not be produced normally. Correspondingly, the symptoms in proband 3 were the most severe in terms of skin and hypotrichosis, whereas proband 2 did not exhibit severe dry skin.

In conclusion, we investigated the genetic and clinical features of patients with XLHED. Three variants located in or affecting exon 1 of the *EDA* gene were identified, including two novel variants of the splicing donor site (c.396 + 1 G > C) and c.171-173 del GTT. We further demonstrated the role of c.396 + 1 G > C in altering gene transcription (creating a cryptic 5′ splice site in exon 1) *in vitro*, which facilitates accurate prenatal diagnosis and genetic counseling for other family members of pedigree 1. As there is still no effective treatment for XLHED, our findings also broaden our knowledge of the *EDA* gene in HED patients, and can be used as a reference for clinical disease screening, diagnosis, and genetic counseling.

## Data Availability

The datasets for this article are not publicly available due to participant/patient anonymity. Requests to access the datasets should be directed to the corresponding author.
